# Several New Putative Bacterial ADP-Ribosyltransferase Toxins Are Revealed from In Silico Data Mining, Including the Novel Toxin Vorin, Encoded by the Fire Blight Pathogen *Erwinia amylovora*

**DOI:** 10.3390/toxins12120792

**Published:** 2020-12-11

**Authors:** Olivier Tremblay, Zachary Thow, A. Rod Merrill

**Affiliations:** Department of Molecular and Cellular Biology, University of Guelph, Guelph, ON N1G 2W1, Canada; otrembla@alumni.uoguelph.ca (O.T.); zthow@uoguelph.ca (Z.T.)

**Keywords:** mono-ADP-ribosyltransferase toxins, *Erwinia amylovora*, toxin-antitoxin, bacterial toxins, agriculture diseases

## Abstract

Mono-ADP-ribosyltransferase (mART) toxins are secreted by several pathogenic bacteria that disrupt vital host cell processes in deadly diseases like cholera and whooping cough. In the last two decades, the discovery of mART toxins has helped uncover the mechanisms of disease employed by pathogens impacting agriculture, aquaculture, and human health. Due to the current abundance of mARTs in bacterial genomes, and an unprecedented availability of genomic sequence data, mART toxins are amenable to discovery using an *in silico* strategy involving a series of sequence pattern filters and structural predictions. In this work, a bioinformatics approach was used to discover six bacterial mART sequences, one of which was a functional mART toxin encoded by the plant pathogen, *Erwinia amylovora*, called Vorin. Using a yeast growth-deficiency assay, we show that wild-type Vorin inhibited yeast cell growth, while catalytic variants reversed the growth-defective phenotype. Quantitative mass spectrometry analysis revealed that Vorin may cause eukaryotic host cell death by suppressing the initiation of autophagic processes. The genomic neighbourhood of Vorin indicated that it is a Type-VI-secreted effector, and co-expression experiments showed that Vorin is neutralized by binding of a cognate immunity protein, VorinI. We demonstrate that Vorin may also act as an antibacterial effector, since bacterial expression of Vorin was not achieved in the absence of VorinI. Vorin is the newest member of the mART family; further characterization of the Vorin/VorinI complex may help refine inhibitor design for mART toxins from other deadly pathogens.

## 1. Introduction

Several important bacterial pathogens encode mono-ADP-ribosyltransferases (mARTs) as secreted toxins that ADP-ribosylate a variety of eukaryotic target macromolecules in order to interfere with essential cellular functions [1]. These bind NAD^+^ and covalently transfer an ADP-ribose moiety to a target protein or DNA molecule in order to modify its activity [2]. Bacterial mARTs are implicated in notorious human diseases such as cholera and diphtheria, and new toxins have recently been recognized for their role in agriculturally important diseases of plants and insects [3,4,5,6].

The mART toxin reaction mechanism is conserved, but the substrate targets are diverse and traditionally are the basis for classification of subgroups [7,8]. Members of the DT (diphtheria toxin)-group ADP-ribosylate elongation factor 2 (eEF2), which inhibits the eEF2-catalyzed translocation of peptidyl-tRNA along mRNA during translation [9,10,11]. CT (cholera toxin)-like mART toxins target heterotrimeric G-proteins to interfere with cAMP signalling by ADP-ribosylating the α-subunit of the signalling protein Gs, trapping adenylyl cyclase in an active state, leading to an intracellular accumulation of cAMP and the subsequent hyper-activation of chloride ion channels via cAMP-dependent protein kinase [12,13]. The CT-like group is further subdivided into C2 and C3 toxins which both act to unfavourably redistribute actin within host cells: members of the C2-group ADP-ribosylate globular actin at Arg-177, which prevents polymerization into filamentous actin through steric hindrance and actin capping, while C3-group toxins ADP-ribosylate GTP-binding proteins Rho A, B and C at Asn-41 to inhibit Rho protein activation and signalling [14,15,16,17]. In addition to the protein-targeting mART toxins, bacterial DNA-targeting mARTs have been identified. They are classified as members of the Pierisin group of mARTs, which are most closely related to the CT-like group. These toxins non-selectively transfer ADP-ribose to 2′-deoxyguanosine in double-stranded DNA, causing the formation of genotoxic DNA adducts [18,19].

Structurally, the enzymatic A-domain of bacterial mARTs is conserved but, like substrate specificity, the mechanism of host cell delivery varies between different toxins. DT-like group toxins and C2-like toxins assume an AB-dimer domain organization where the A-domain is delivered to the host cell cytoplasm and exhibits cytotoxic ADP-ribosyltransferase activity, and the B domain is responsible for receptor-binding leading to translocation and intracellular delivery of the A component [20,21,22,23]. Translocation of CT-like toxin A-domain is accomplished in a similar manner to DT-like toxins, where a B-subunit pentamer forms the receptor-binding domain [24,25]. In contrast, C3-like toxins are A-only and enter host cells autonomously via a currently unknown mechanism [26].

Overall sequence homology among all members of the mART toxin family is low, as sequence identity within any given group ranges from 29% to 64%, and as low as 15% between any two members from different groups; nonetheless, a common mART three-dimensional core fold structure and three important conserved catalytic regions involved in NAD^+^ binding are present in all members of the CT-, C2-, and C3-groups [27]. The conserved catalytic core that forms the NAD^+^ binding pocket is characterized by a β/α fold, where two perpendicular β-sheets are followed by one α-helix in C2- and C3-like toxins, and two α-helices in the other groups, which makes up the cleft where the nicotinamide ring of NAD^+^ is bound during catalysis [28,29]. The region 1 motif of CT-, C2- and C3-like mART toxins is located on β1, and contains an aromatic residue, usually Tyr or Phe, followed by a conserved Arg that creates a hydrogen bond with a phosphate oxygen of NAD^+^ [28,29]. Region 2 comprises a Ser-Thr-Ser (STS) motif located at the end of β3 which plays a role in orienting NAD^+^ in the active site but also a structural role in stabilizing the binding pocket by connecting the perpendicular β-sheets [28,29]. The second serine in the STS motif is replaced by either a Thr or Gln residue in some toxins [30]. Region 3, termed the ADP-ribosylating turn-turn (ARTT) motif, is located between β5 and β6, and contains a [QE]-x-E motif where the second, solvent-exposed Glu residue forms a hydrogen bond with N-ribose of NAD^+^, and is involved in transferase activity in all mART toxins [27,29]. The presence of a Glu or Gln residue in the E(Q)-x-E motif of C2 mARTs and C3 mARTs, respectively, appears to play a role in substrate specificity between actin and Rho proteins, as Gln is required for ADP-ribosylation of Asn-41 [31,32]. Facing the ARTT loop, the phosphate-nicotinamide (PN) loop stabilizes the nicotinamide phosphate during binding of NAD^+^_,_ and is located immediately after the STS motif between β3 and β4 [33]. To functionally relate mARTs by primary sequence, a regular expression encompassing the conserved motifs has been derived for all CT-, C2, and C3-group toxins:

[YFL]-R-x(27,60)-[YF]-x-S-T-[SQT]-x(32,78)-[QE]-x-E [30]

**Figure 1:**
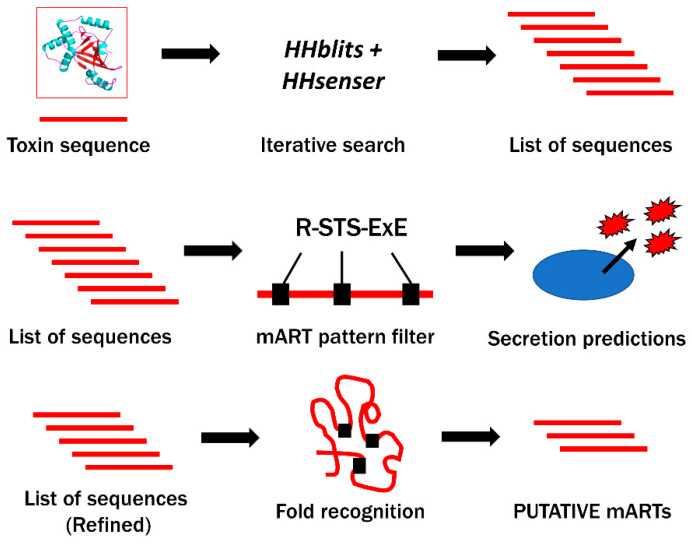
Summary of mono-ADP-ribosyltransferase (mART) toxin discovery pipeline.

However, inferring homology from a functional family of proteins with such remarkably low sequence identity is a challenging task which requires an elegant and efficient set of bioinformatics resources. Despite low sequence identity, the conserved three-dimensional catalytic core fold is amenable to a structure-based data mining approach with which remote homologs may be discovered [27,28]. This strategy was described by our research group in 2008 and applied in 2010, unveiling six new bacterial mART toxins, some of which were from human pathogens such as *Vibrio cholerae* and *Enterococcus faecalis* [27,30]. More recently, also owing to the data mining efforts reported by Fieldhouse et al. in 2010, our lab has reported biochemical data on the agriculturally relevant mART toxin Scabin, produced by the soil-dwelling potato pathogen, *Streptomyces scabies,* and the honey bee toxins Plx2A and C3larvinA, both produced by the causative agent of American Foulbrood disease, *Paenibacillus larvae* [5,6,34]. Briefly, our bioinformatics pipeline (summarized in Figure 1) involves mining databases like UniProt and the National Center for Biotechnology Information (NCBI) for homologous protein sequences, then applying sequential filters to extract sequences with mART characteristics.

Using the protein sequence of a known mART toxin as input, a list of several thousands (10,000–20,000) of candidate protein sequences is created using iterative search tools such as HHblits and HHsenser, analogous to a large-scale BLAST search that is catered to protein homologs with low sequence identity [35,36]. Next, the sequences are subjected to pattern-based filters, including searches for the conserved mART motif, followed by predictions for the absence of transmembrane domains, and the presence of secretion signal peptides or other indicators of non-classical secretion. Finally, consensus fold recognition analyses determine whether the remaining sequences are likely to assume a mART-like structure by generating homology models. The final list represents putative mART toxin sequences that are tested in the lab according to a yeast growth deficiency assay that was developed in our lab to validate computer predictions [37]. As antibiotic resistance becomes increasingly problematic in the treatment of bacterial diseases, inhibitor-based anti-virulence strategies become more appealing as they aim to disarm pathogens rather than kill them and, therefore, do not impose as much selective pressure [38,39]. The identification of novel mART toxins has contributed to the design of new inhibitors with therapeutic potential relevant to healthcare and agriculture [5,40,41]. Hence, the ongoing discovery of novel mART toxins by exploiting burgeoning genomic libraries will ultimately diversify our repertoire of target virulence factors for inhibitor design and could improve design strategies by unveiling new common structural or biochemical features among the different mART groups. The discovery of new virulence factors also contributes to a better understanding of bacterial diseases for which the mechanism of pathogenesis is currently uncharacterized. More than 240,000 prokaryote genome sequences have currently been submitted to NCBI (https://www.ncbi.nlm.nih.gov/genome/browse#!/prokaryotes/); the current status of sequence data availability, coupled with the proven abundance of mARTs in bacterial genomes, creates an environment where new mART toxins are once again ripe for discovery.

Herein we describe our latest genome sequence database search for new mART toxins using an in silico method established by our lab and provide a summary of several new potential mART virulence factors. We also present a preliminary characterization of a new toxin of consequence to agriculture as evidence for its inclusion in the mART family: Vorin, produced by the fire blight pathogen *Erwinia amylovora.*

## 2. Results and Discussion

### 2.1. Bioinformatics Genome Mining and Candidate Mono-ADP-Ribosyltransferase (mART) Sequence Filtering

Remote homologs representing putative mART toxin candidates were retrieved by performing sequence searches of UniProt and NCBI databases using HHsenser and HHblits. The list of all sequences obtained from remote homology searches, which includes sequences from all 18 individual datasets, contained a total of 12,993 protein sequences inferred from genomic sequencing data found in the UniProt, UniParc, and NCBI databases. Once duplicate sequences, sequences with more than 1000 residues, and sequences that did not match the mART regular expression were discarded, the list was reduced to 229 candidates. The steep reduction of sequences to <2% of the original total was in part due to the convergence of search results, as independent output lists contained duplicate UniProt identifiers. Following secretion and transmembrane domain predictions, the list was further reduced to 160 probable mART sequences that were likely secreted, and not associated to the cell membrane or bacterial cell wall. These 160 sequences were then ranked according to novelty and virulence, as only sequences with favourable genomic context produced by pathogenic bacteria were considered for structure-based filtering. The genomic neighbourhood of predicted mART toxin sequences was examined for the presence of gene products predicted to contribute to virulence, including accessory toxin genes, genes encoding toxin secretion machinery, or general hallmarks of genomic islands like integrase genes, transposable elements, and tRNA genes [42]. Of the sequences that met the genomic criteria, only those with less than 50% sequence identity to previously characterized mART toxins were kept, reducing the final sequence candidate count to 16 for structural modeling.

A consensus approach was applied using Local Meta-Threading Server (LOMETS) to assess whether multiple structure prediction servers agreed that the remaining 16 catalytic domain sequences assumed a mART-like fold (Table 1). Only two of the 16 sequences, A0A093SYU3 and E0LU50, did not meet the 7/10 server threshold, indicating that in those two cases a common three-dimensional structure prediction was not confidently achieved. Sequence D0Z4Y1 from *P. damselae* showed at least 7 out of 10 servers predicting a mART fold, however the template coverage was below 70%, and the sequence was, therefore, discarded. Sequence E5B8T9 from *E. amylovora* showed the lowest template coverage (0.510) but displayed a perfect high confidence server prediction score of 10. E5B8T9 was not discarded since it was the same C-terminal half of the sequence that showed a high prediction confidence according to each server result, revealing that the input amino acid sequence contained a second functional N-terminal domain.

A threading-based prediction approach using RaptorX (Xu Group, University of Chicago, Chicago, IL, USA) was then applied to the 13 remaining protein sequences to obtain quantifiable indicators of model quality, and to determine which characterized mART toxin structures serve as the best threading templates (results summarized in Table 2). SeqID scores were considered for ranking purposes, but not for eliminating any putative mARTs as mART toxin sequence identity is characteristically low [27]. If a model was generated using mART toxin structures as templates and if the scores for a given model met each of the minimum recommended cut-offs, then the putative mART toxin was considered to adopt a mART-like tertiary structure.

Sequences A0A0W0XJR5 and W3YC38 were discarded since their GDT scores were less than 50, suggesting that only certain portions of the model were able to be correctly threaded to a mART toxin structural template. Sequence W3YC38 was also removed due its borderline *p*-value of 1.00 × 10^−4^. The other 11 sequences displayed values within the accepted threshold for each scoring parameter matched to mART toxin structural templates from the PDB. The non-mART toxin structure 4ELN, an unpublished type III effector from the plant pathogen *Xanthomonas axonopodis* pv. *citri*, was identified as a threading template for sequence T9AJP6 [43]. All other PDB IDs listed as templates in Table 2 correspond to experimentally determined mART toxin structures.

The final list of 11 candidate mART toxins was further reduced to six; a number that could feasibly be validated in the yeast growth deficiency assay. Factors pertaining to wet lab amenability such as protein expression and solubility were considered, but these six putative mARTs were also subjectively prioritized based on the research interests of our laboratory. Although only a final group of six putative toxins is described in this work, it is worth noting that the remaining 11 sequences, and possibly even more sequences further up the chain of bioinformatics filters, are still potential mART toxins. The list was filtered and refined using stringent criteria at the expense of missing bona fide toxins. A short description of five new putative mART toxins follows below, summarizing any biological or biochemical properties predicted from our bioinformatics analysis. A preliminary characterization of a new mART toxin is also presented. A summary of each of these toxins is outlined in Table 3.

For some UniProt accession IDs, high percent identity (90%+) homologs exist; the IDs shown correspond to the sequence originally mined by remote homology searches. Percentages shown correspond to sequence percent identity as calculated by pairwise UniProt alignment of catalytic domains.

### 2.2. Bovin

Bovin is a putative mART toxin encoded by a bovine isolate of the fastidious Gram-negative bacterium, *Bartonella bovis. B. bovis* has been shown to cause endocarditis and persistent bacteremia in cattle [44,45]. A phylogenetic survey of several pathogenic *Bartonella* species revealed that flagellin is likely a major virulence factor of *B. bovis*; however, secreted virulence factors have not yet been identified [46]. Bovin may represent a deadly tool used by *B. bovis* during infection.

**Figure 2:**
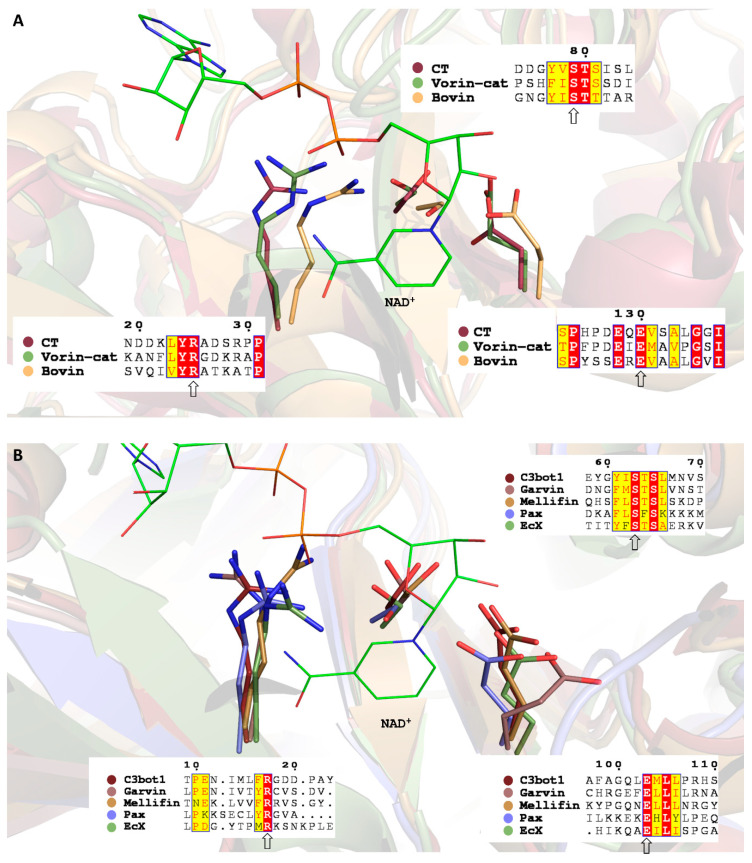
Active site view of model superpositions with Cholera toxin and C3bot1. (**A**) Superposition of predicted CT-like toxin models with the NAD^+^-bound structure of Cholera toxin. (**B**) Superposition of predicted C3-like toxin models with the NAD^+^-bound structure of C3bot1. Catalytic glutamate residues in the structure of CT have been substituted with aspartate residues. Sequence alignments were undertaken with T-COFFEE, using the Expresso structural alignment option.

**Figure 3:**
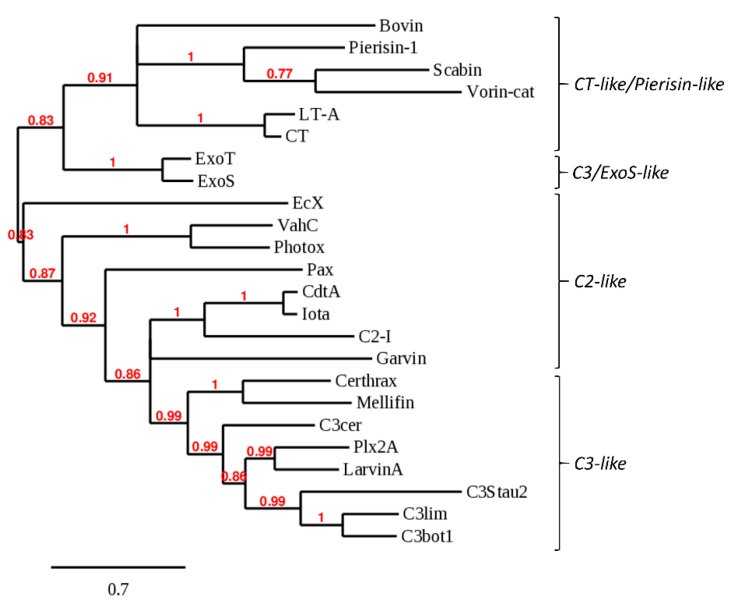
Phylogenetic relationship of new putative mARTs with characterized mART toxins. Branch support values are shown in red. Phylogenetic tree was generated using Phylogeny.fr by T-COFFEE alignment and Bayesian inference.

Bovin is a single-domain, CT/Pierisin-like protein that exhibits relatively high sequence identity with cholera-like toxins and with the DNA-targeting toxins Pierisin-1 and Scabin. Bovin possesses an R-STS-E(Q)xE mART motif, where all residues are positioned in the mART active site in an identical fashion to cholera toxin (Figure 2A). Bovin also has Trp and Lys residues equivalent to Trp128 and Lys130 in Scabin that have been proposed to interact with DNA [47]. Lys130 is unique to Scabin and Bovin, while Trp128 is unique to DNA-targeting mARTs. However, in contrast to Scabin and Pierisin-1, Bovin has an ExE catalytic motif rather than a QxE motif, which may preclude Bovin from being a DNA-targeting toxin. The closest phylogenetic relatives of Bovin are Pierisin-1 (Figure 3). Secondary structure predictions revealed that Bovin may possess a pentameric B-domain for toxin delivery like cholera toxin and LT, as a short peptide which has a propensity for forming an α-helical structure like cholera toxin B subunits is encoded directly downstream of Bovin. Bovin did not elicit a cytotoxic effect in the yeast growth deficiency assay; this may lend to the possibility that DNA is the target substrate for Bovin since it would not be able to access the nucleus or mitochondria of *S. cerevisiae* to target DNA for ADP-ribosylation in the absence of a putative translocation domain. Bovin is potentially the second DNA-targeting mART toxin secreted by a bacterial species.

### 2.3. EcX

EcX is a putative mART toxin encoded by a human blood isolate of the Gram-negative bacterium *Enterobacter bugandensis,* which is part of the *Enterobacter cloacae* complex. Species of *Enterobacter* are increasingly problematic because they are multi-drug resistant nosocomial pathogens that cause infections such as septicemia, pneumonia, osteomyelitis, urinary tract infections, and intra-abdominal infections [48,49]. Virulence of *Enterobacter* spp. is currently attributed to T3SS effectors, curli fimbriae, siderophores, and several drug-resistance mechanisms [48,49].

EcX is a 951-residue protein, with a distinct mART domain (residues 679–951), and a large N-terminal domain that may serve as a toxin delivery and encapsulation structure analogous to the BC component of Tc from *P. luminescens* (PDB 409X) and the Rhs-repeat containing the BC component of ABC toxin from *Yersinia entomophaga* (PDB 4IGL) [50,51]. The two domains are delimited by a Factor Xa protease recognition site (IEGR); proteolytic cleavage may, therefore, be responsible for toxin release upon delivery into the host cell. The closest mART homologs of the predicted catalytic domain of EcX are Mav and Vis, which share 27% and 22% sequence identities, respectively. The mART catalytic signature of EcX includes a QxE motif (Figure 2B), which may suggest that asparagine is the target residue like with Rho-targeting C3-like mARTs. However, the closest phylogenetic relatives of EcX were VahC and Photox, which are ExE-motif C2-like toxins (Figure 3). EcX was not tested in the yeast growth deficiency assay as no source of DNA encoding the putative toxin was obtained. Regardless, EcX may represent an important secreted effector that contributes to the severe pathogenic potential of *E. bugandensis* [48].

### 2.4. Mellifin

Mellifin is a putative mART toxin encoded by *Spiroplasma melliferum*, a bacterial species that has been shown to infect honey bees and has been isolated from their feces, but that has not yet been proven as a honey bee pathogen [52,53]. Nonetheless, the identification of a mART toxin produced by *S. melliferum* may inspire further research into potential pathogenic interactions with honey bees.

Mellifin shares a 42% sequence identity with the toxin Certhrax, which is a mART that, like anthrax lethal factor, requires protective antigen (PA) for cell entry [41]. Certhrax harbours a PA-binding domain, but Mellifin does not; instead, Mellifin has an extended N-terminal helix that is uncharacteristic of single-domain C3-like mARTs but is too short to comprise a putative PA-binding domain. This finding is misleading, as a protective antigen homolog is encoded directly downstream of Mellifin. The N-terminal extension of Mellifin may be a vestigial remnant of a once complete PA-binding domain, as a genomic investigation of the closely related species, *S. citri,* revealed that 21% of chromosomal coding sequences were truncated compared to predicted homologs [54]. A comparable amount of gene decay in *S. melliferum* may be responsible for the reduced remnants of a Mellifin PA-binding domain. The relationship of Mellifin to characterized mART toxins was also ambiguous, as it did not show any distinct phylogenetic grouping. Like Bovin, Mellifin did not cause a cytotoxic effect in the yeast growth-deficiency assay and may require a host-specific factor for enzyme activation.

### 2.5. Pax

Pax is encoded by *Parachlamydia acanthamoebae*, which is a Gram-negative obligate intracellular bacterium [55]. It naturally infects free-living amoebae, and is capable of resisting destruction by human macrophages, likely by controlling vacuole formation [56]. *P. acanthamoebae* is of interest as a pathogen because of the difficulty associated with aetiological diagnosis of community-acquired pneumonia, and may represent a significant cause of this disease [57]. *P. acanthamoebae* may deploy T3SS effector proteins to modulate pathogen-containing vacuole biogenesis [56]. Indeed, the same strain that encodes Pax encodes a T3SS homolog; however, they are located on separate contigs of an unassembled genome, so it is difficult to assess their proximity.

Pax is a mART homolog of EFV and Vis toxins. The predicted structure of Pax included a 41-residue N-terminal sequence that could not be successfully modeled to any template, and incidentally, for which no homologs existed. No clear phylogenetic mART grouping was able to be identified. At every stage of in silico filtering, discarded sequences were occasionally re-evaluated to ensure they were correctly omitted. Upon re-examination, it was noted that the only filter that Pax did not pass was the mART regular expression due to a unique R-SFS-ExE motif, where all motif elements were still permissibly spaced within the limits of the regular expression, but an SFS motif violated the strict S-T-[STQ] motif condition of the expression. Despite the SFS motif, all residues from the catalytic motif were correctly positioned in the active site (Figure 2B). Pax was kept as a finalist putative mART to challenge the mART motif template if Pax demonstrated the ability to elicit a cytotoxic effect upon expression in *S. cerevisiae*. However, Pax was the second putative mART toxin that was not able to be tested in the yeast growth deficiency as no genetic material encoding Pax was obtained.

### 2.6. Garvin

Garvin is a 27.5 kDa, 239-residue single-domain protein encoded by a human septicemia isolate, strain 21881, of the pathogen *Lactococcus garvieae*. *L. garvieae* is a Gram-positive pathogen responsible for causing lactococcosis, a haemorrhagic, often fatal disease affecting freshwater and marine fish, notably salmonid species like rainbow trout [58]. Lactococcosis poses an economic burden to the European aquaculture industry which produces 100,000 tons of fish annually, as infection results in mortality rates as high as 50%, and infected fish that survive become unmarketable due to their decreased weight or due to the unfavourable appearance of symptoms such as exophthalmia or abdominal swelling [59,60]. *L. garvieae* is also considered an emerging human pathogen, since human infections have been associated with septicemia, endocarditis, prosthetic joint infections, UTIs, and skin infections in both healthy and immunocompromised patients [58,59,61].

Unlike any of the fish isolates sequenced to date, *L. garvieae* strain 21881 harbours five circular plasmids, one of which, pGL5, encodes Garvin [62]. Aguado-Urda et al. performed a genetic analysis of the five plasmids, and identified a gene predicted to encode an actin-targeting mART toxin which they have annotated *txn*; incidentally, the bioinformatics data mining performed in this study led to the discovery of this same gene product encoded by *txn,* which was named Garvin [62]. The authors also revealed putative plasmid-encoded virulence factors: these include collagen-binding protein domains, which may endow *L. garvieae* with the ability to bind collagen-rich heart valves [62]. BLAST searches revealed that a strain of *L. garvieae* isolated from mallard duck intestines also encodes a chromosomal homolog of Garvin with 77% sequence identity, and also that at least four homologs of Garvin with sequence identities ranging from 32–46% are encoded by species of *Enterococcus* [63]. It is unclear whether Garvin falls into a C3-like or C2-like grouping (Figure 3); however, its closest mART homolog is C3bot1, with which Garvin shares 24% sequence identity. Garvin is an ExE motif mART toxin, which is more in line with C2-like or ExoS-like single-domain mARTs, and it was therefore surprising that C3bot1, a QxE single-domain mART, showed the highest sequence identity.

### 2.7. Vorin

Vorin is a 31 kDa, 276-residue protein encoded by *Erwinia amylovora* ATCC BAA-2158 (Bb-1, Ea246, IL-5). *E. amylovora* is the causative agent of fire blight, an economically important plant disease that causes exudative cankers in plant hosts from the Rosaceae family [64]. Crop losses combined with treatment costs amount to an estimated $100 million per year in the USA, and still no suitable treatment options exist due to difficulties imparted by the emergence of antibiotic-resistant strains of *E. amylovora*, and limitations imposed on antibiotic use [65,66,67]. The lack of a commercially available control agent with specificity for the bacterial pathogen poses an additional problem in disease treatment [66]. Host pathogenicity of different strains of *E. amylovora* is divided into two distinct groups: the Spiraeoideae (apple, pear) infecting strains and Rubus (raspberry, blackberry) infecting strains, which are rarely capable of cross-infection [68]. *E. amylovora* ATCC BAA-2158 is a Rubus-infecting strain of *E. amylovora* that was originally isolated in Illinois in the early 1970s from a thornless blackberry plant [69].

Vorin-cat is a probable CT-like mART toxin as it possesses an ExE catalytic motif and shares the highest percent sequence identity with the CT-like toxins LT-A and Cholera toxin, and the CT/Pierisin-like DNA-targeting mART toxins, Pierisin and Scabin. Vorin contains two distinct domains: a 14.5 kDa C-terminal catalytic domain which harbours the conserved mART catalytic signature (Vorin-cat), and a conserved 16.5 kDa Rearrangement Hot-Spot (Rhs) N-terminal domain of unknown function (Vorin-NTD). Rhs proteins are a subset of Gram-negative polymorphic toxin systems that are usually large, 1500-residue proteins that undergo type-VI secretion, where the C-terminal domain is a 130–177-residue hypervariable toxin domain encoded by a GC-poor, codon-biased DNA sequence distinct from other Rhs domains [70,71,72]. Separating the two domains is a DPXG-(X_13_)-PXXXXDPXGL Rhs motif [71]. The latter portion of this motif is a predicted auto-proteolytic cleavage site (PXXXXDPXG/L) where a catalytic aspartate dyad from the Rhs motif may be responsible for pH-triggered cleavage and activation of Vorin-cat [50,51]. Consistent with the third and fourth domains of polymorphic Rhs toxins, the gene sequences encoding Vorin-NTD and Vorin-cat each exhibit significant codon bias: the GC-contents of *vorin-NTD* and *vorin-cat* are 62.2% and 39.3%, respectively, while the chromosomal GC-content of *E. amylovora* ATCC BAA-2158 is 53.6% [69].

### 2.8. The Genomic Neighbourhood of Vorin: Type-VI Secretion and Toxin-Antitoxin Modules

**Figure 4:**
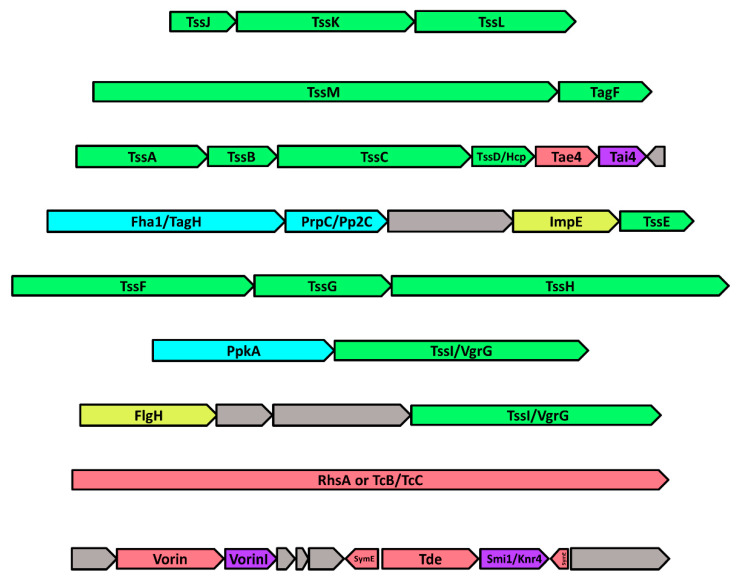
Genomic neighbourhood of *vorin*. Encoded T6SS structural proteins are shown in green, while T6SS regulatory proteins are shown in blue. Aberrant gene products having no definable synergy with the other encoded proteins are shown in yellow. Toxins and antitoxins are shown in pink and purple, respectively. Hypothetical protein open-reading frames (ORFs) with no predicted function are in grey. ORF lengths are to scale, but non-coding regions between ORFs are not depicted. Vorin and VorinI are encoded on the complementary strand of the *E. amylovora* ATCC BAA-2158 chromosome.

A genomic neighbourhood of 44,668 nucleotides encoding 38 predicted ORFs around *vorin* was inspected (Figure 4). A gene cluster encoding a complete set of Type-VI secretion system (T6SS) structural subunit homologs was identified upstream of Vorin where all 13 required T6SS proteins were successfully mapped. The membrane complex is composed of subunits TssJ, TssL, and TssM, which form 10 heterotrimeric structures that span the bacterial inner and outer membranes to anchor the T6SS [73]. The baseplate complex forms on the cytosolic side of the inner membrane, and comprises subunits TssA, TssE, TssF, TssG, TssK and TssI which serve as a scaffold for assembly of the T6SS contractile sheath, and for assembly of the hexameric Hcp/TssD tunnel structure through which effectors are deployed [73]. The T6SS sheath that generates the contractile force required for injection is composed of small subunit TssB and large subunit TssC, and these subunits are then recycled by the ATPase TssH/ClpV after contraction [73]. The presence of a complete host of T6SS genes upstream of *vorin* suggests that Vorin is a type-VI-secreted toxin. Two proteins which are likely involved in the regulation of T6SS assembly were also identified. The putative serine/threonine protein kinase PpkA is encoded near the end of the T6SS cassette, and further upstream, a putative PrpC- or Pp2C-family serine/threonine phosphatase is encoded adjacent to the T6SS-associated inner-membrane protein Fha1. PpkA has been shown to phosphorylate Fha1 at a threonine residue to promote T6SS assembly, while a different phosphatase, PppA, dephosphorylates Fha1 to negatively regulate assembly [74,75]. In the Vorin T6SS gene cluster, the PrpC/Pp2C phosphatase may adopt a similar role to PppA. Repression of T6SS assembly has also been shown to involve an interaction between Fha1 and the T6SS accessory protein TagF in the plant pathogens *Agrobacterium tumefaciens* and *P. aeruginosa* [76]. *E. amylovora* ATCC BAA-2158 likely possesses both mechanisms of assembly/disassembly as a TagF homolog is also encoded in the Vorin T6SS cluster between TssM and TssA (Figure 4).

Effector and Rhs proteins secreted by Type VI-secretion systems (T6SS) are usually implicated in bacterial competition with neighbouring cells both at the inter- and intra-specific level, and are often encoded near or adjacent to cognate immunity proteins that protect the attacking organism against autointoxication [77]. Typically, toxin-antitoxin systems have been reported in Gram-negative pathogens wherein a highly toxic T6SS effector protein is bound directly by a specific, immunity-conferring antitoxin [77,78]. In *S. marcescens* Db10 co-culture experiments, the secreted effectors Rhs1 and Rhs2 are responsible for T6SS-dependent killing of mutant strains deficient in the cognate immunity partners RhsI1 and RhsI2, while mutant strains complemented with RhsI1 and Rhs2I were unaffected by the toxins [79]. The same protective effect was observed when the *D. dadantii* Rhs toxins were expressed in *E. coli* with their antitoxin RhsI [80]. Other Rhs effectors possessing cognate immunity proteins, including metallopeptidase and deaminase toxins from *E. coli* and an Rhs toxin with uncharacterized antibacterial activity from *P. aeruginosa*, have been identified [81,82].

Three distinct toxin-antitoxin modules were predicted in the Vorin T6SS cassette. Homologs of the type-VI secreted effector Tae4, an amidase that degrades bacterial cell wall peptidoglycan, and Tai4, its cognate immunity protein, are encoded in the middle of the T6SS subunit homologs [83]. Next, a homolog of the type-VI secreted deoxyribonuclease toxin Tde from *A. tumefaciens* is located just downstream of Vorin. Tde is involved in interbacterial competition against *P. aeruginosa* and other strains of *A. tumefaciens* [84]. However, no homolog of its cognate antitoxin Tdi was identified; instead, a Smi1/Knr4 family protein is found immediately downstream of the Tde homolog. Smi1/Knr4 was originally characterized in *S. cerevisiae* as a protein that conferred resistance to toxin secretion by other antagonistic yeast, but a bioinformatics study performed by Zhang et. al. revealed that Smi1/Knr4 homologs in bacteria are potential immunity proteins for nuclease effectors [85]. In this scenario, Smi1/Knr4 could be acting as an antitoxin for the adjacently encoded Tde DNase homolog. The third type of TA module identified comprises two genes encoded downstream of Vorin on the reverse strand that encode predicted SymE family toxins from the SymE-SymR toxin-antitoxin system. SymE is a toxin that cleaves cellular mRNA to inhibit translation, and belongs to a type I toxin-antitoxin module [86]. Its antitoxin, SymR, is a cis-encoded, antisense non-coding RNA that base-pairs with the toxin mRNA transcript, preventing production of SymE [87]. SymE is possibly implicated in recycling of damaged RNA as part of the SOS response [87]. Given that an endonuclease toxin is encoded directly adjacent to the predicted SymE genes, the SymE/SymR TA module in this genomic context may serve as a requisite preservation mechanism, whereby SymE activity is induced under stress conditions to counter any sudden DNA or RNA damage caused by inadvertent self-intoxication.

A number of parallels exist between the Vorin/T6SS gene cluster and the T6SS/Rhs protein gene cluster of *Serratia marcescens* Db10 reported by Diniz and Coulthurst, namely the presence of all 13 T6SS proteins, nearly identical T6SS kinase and phosphatase regulatory proteins, and Tae/Tai-family TA modules [79]. Given these similarities, in a manner analogous to the Rhs1 and RhsI1 modules, Vorin was predicted to possess a cognate antitoxin in the same position as RhsI1 on the basis that the two T6SS gene clusters were highly conserved. BLASTp searches did not indicate that this ORF encoded a Vorin antitoxin, but the gene adjacently downstream to *vorin* in Figure 4 is annotated as VorinI (Vorin Immunity protein) because its role as an antitoxin is experimentally described in this study. In contrast to the *S. marcescens* counterpart Rhs1, the N-terminal Rhs domain of Vorin is significantly truncated to only ~11% of the length of the Rhs1 N-terminal domain (143 residues vs. 1332 residues). The TcB-TcC homolog (1540 residues) may compensate for this truncation in *E. amylovora* ATCC BAA-2158, as it is absent in the *S. marcescens* Db10 T6SS cluster, but the mechanism for which it might compensate is unknown.

The largest ORF in the Vorin T6SS gene cluster is a homolog of both the DNase toxin RhsA from *Dickeya dadantii* and of the BC encapsulation shell structure from the ABC Rhs toxin Tc produced by *P. luminescens* [50,70]. Although depicted as an Rhs family toxin gene in Figure 4, no catalytic domains are predicted within the ORF, meaning it probably only encodes a conserved Rhs structural element involved in secretion or translocation to the host. TcB and TcC form a large protective shell in which the mART toxin catalytic TcA domain is sequestered prior to secretion to prevent autointoxication, and then likely undergoes autoproteolysis triggered by pH acidification inside the host insect midgut [50]. The TcB-TcC homolog may represent an alternative delivery method for Vorin, but Vorin is adjacent to an immunity protein so the TcB-TcC homolog is likely not the primary mechanism of secretion or of protection from autointoxication, but rather involved in translocating other potential effectors encoded nearby that lack a cognate antitoxin. Gene knock-out studies would be required to determine which effectors depend on the T6SS for secretion, and which effectors, if any, are secreted by a mechanism like Tc toxin.

### 2.9. Vorin and Garvin Yeast Growth Deficiency Assays: Validation of Bioinformatics Predictions

**Figure 5:**
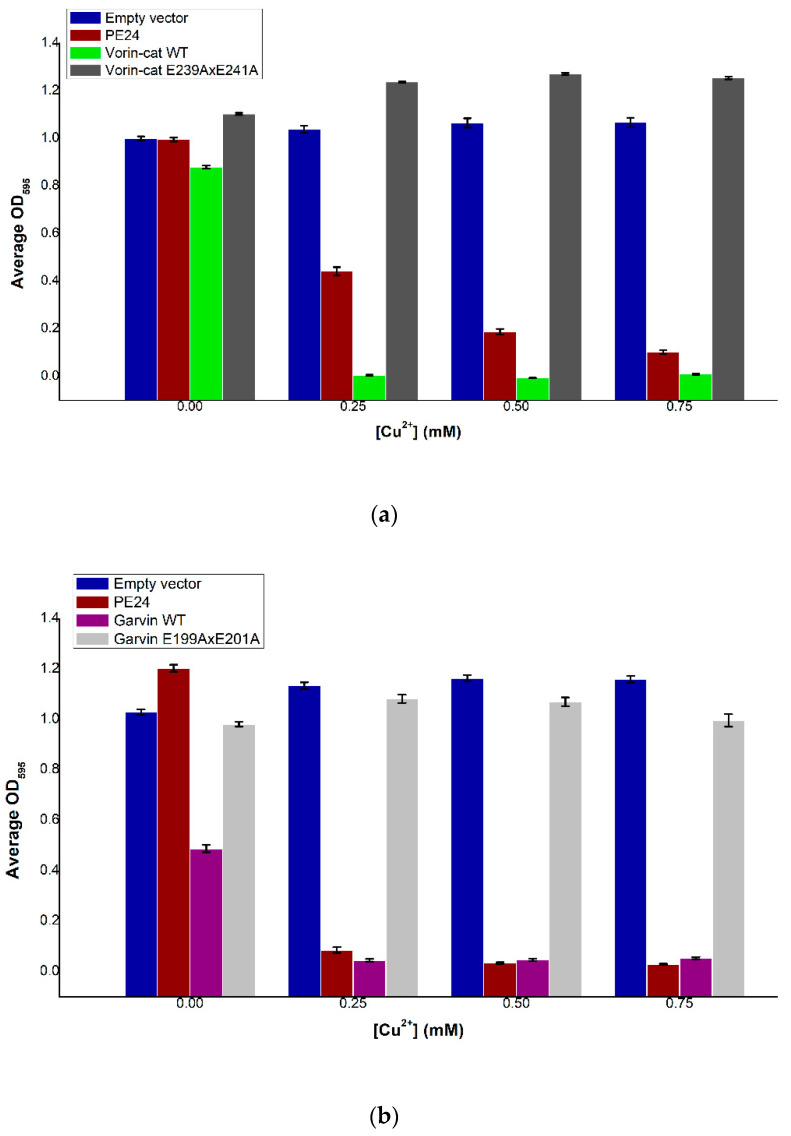
Yeast growth deficiency assays. Cells were transformed with either empty pRS415-*CUP1* plasmid, pRS415-*CUP1-vorin-cat*, or pRS415-*CUP1-garvin* and expression was induced with increasing amounts of Cu^2+^ inducer for 48 h. Data shown are the average of three experiments comprising at least four biological replicates, each consisting of four technical replicates ± standard error. PE24, the catalytic domain of the well-characterized mART toxin, ExoA from *P. aeruginosa*, was expressed as a positive control for the yeast growth-defect phenotype.

Wild-type Vorin-cat was expressed in vivo in *S. cerevisiae* under the control of the copper-inducible promoter *CUP1* to determine its cytotoxic potential. Induction of Vorin expression resulted in a dose-dependent decrease in yeast cell culture densities compared to empty vector controls after 48 h of incubation (Figure 5a). Growth of cells expressing Vorin-cat WT was severely impaired at all concentrations of Cu^2+^, while uninduced Vorin-cat WT cultures elicited only a minor growth defect. Moreover, cell densities of yeast expressing Vorin-cat WT were lower than those expressing PE24 (positive control) at all concentrations of Cu^2+^. To confirm that the observed cytotoxic effects could be ascribed to the expression of Vorin-cat WT toxin, the essential catalytic glutamate residues responsible for transferase activity in mART toxins were substituted with alanine residues via site-directed mutagenesis (Vorin E239AxE241A). When the experiment was repeated with Vorin E239AxE241A, cell growth resembled that of empty vector control cultures, indicating that the growth-deficiency phenotype was reversed, and that cytotoxicity was due to the enzymatic activity of Vorin-cat WT. Given that Vorin-cat WT could elicit a cytotoxic effect in a eukaryotic yeast model, in conjunction with the presence of a predicted proteolytic cleavage site, Vorin-cat is hypothesized to represent the biologically active version of this mART enzyme.

Induction of Garvin expression resulted in a significant reduction of yeast cell culture densities at all concentrations of Cu^2+^ compared to empty vector controls after 48 h of incubation (Figure 5b). Cell densities of yeast that contained pRS415-*CUP1*-*garvin,* but where Garvin was not induced, also elicited a growth defect as the average culture optical density was approximately half of the empty vector control culture at 0 mM Cu^2+^. This result suggests that even minimal background expression levels of Garvin were sufficient to cause a marked reduction in yeast cell viability. When the experiment was repeated after substituting essential catalytic glutamate residues with alanine residues via site-directed mutagenesis (Garvin E199AxE201A), cell growth was similar to that of empty vector control cultures, indicating that the growth-deficiency phenotype was due to the enzymatic activity of wild-type Garvin.

### 2.10. Quantitative Liquid Chromatography–Tandem Mass Spectrometry (LC-MS/MS) of Cells Expressing Vorin-Cat: A Possible Role in the Suppression of Autophagy

**Figure 6:**
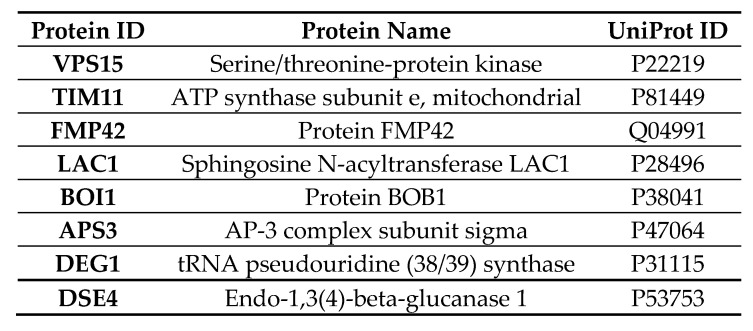

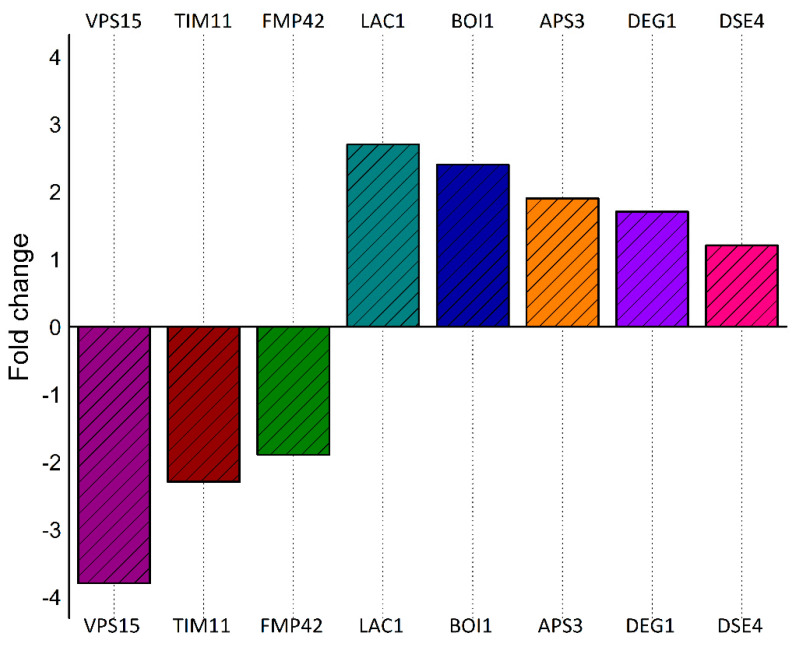
Change in abundance of *S. cerevisiae* proteins resulting from expression of Vorin-cat. UniProt protein names and their corresponding accession ID are listed. Fold change indicates the relative abundance of each protein compared to yeast cells that were transformed with Vorin-cat expression vector, but where expression of Vorin-cat was not induced.

Mass spectrometry analysis was performed to measure significant changes in the proteome of *S. cerevisiae* after expression of Vorin-cat was induced, either through a positive- or negative- enrichment of proteins compared to an uninduced sample set. A total of 2450 proteins were detected from the 6049 proteins in the *S. cerevisiae* s288c reference proteome. The Vorin-cat sequence was manually added to the reference proteome database, but no Vorin-cat derived peptides were detected, likely because their abundance was below the limit of detection for the parameters of the experiment. However, the expected toxic effect of Vorin-cat was observed in induced yeast cultures as they showed a drastically reduced cell density relative to uninduced controls before processing of both culture sets. Nonetheless, a summary of the eight proteins that showed a statistically significant change in abundance is presented in Figure 6. Three proteins were negatively enriched upon Vorin-cat expression, while five proteins were positively enriched.

Protein VPS15 showed a 3.8-fold decrease in abundance compared to the control sample set, which was the highest magnitude of change observed for any protein. In yeast, VPS15 is a 150 kDa protein with a putative serine-threonine kinase domain that, along with VPS34, is vital to the formation of kinase complexes I and II, which are responsible for autophagy initiation, and for endosomal sorting and maturation, respectively [88]. In both complexes, VPS34 acts as a PI3 kinase, and is required for vacuolar protein sorting by producing PI3P which recruits downstream effectors to endosomal compartments [89,90]. VPS15 has recently been shown to allosterically inhibit VPS34 activity in an artificial subunit tethering experiment; however, formation of the complete VPS34-VPS15-ATG14-BECN1 complex I structure was shown to be essential for VPS34 kinase activity as synthesis of PI3P was almost completely abolished when VPS34 was isolated compared to when it was part of the fully-assembled complex [90]. VPS15 also plays a role in recruiting the acid hydrolase carboxypeptidase Y (CPY) from the Golgi to vacuoles during autophagy [91]. Interestingly, yeast mutants lacking VPS15 and VPS34 were shown to exhibit impaired transcription elongation and reduced mRNA production from G + C-rich coding sequences [92]. Autophagy in both yeast and mammals involves autophagosomal degradation and subsequent recycling of protein aggregates and damaged organelles via lysosomal acidification and enzymatic hydrolysis, and is also implicated in the clearance of intracellular pathogens [93]. Dysregulation of autophagy often leads to cell death and has been associated with disease in humans such as Parkinson’s disease [93,94]. Expression of Vorin-cat in yeast caused a nearly 4-fold decrease in VPS15; as VPS15 has been shown to be essential for autophagy initiation, Vorin-cat may suppress autophagy in yeast, and subsequently cause an accumulation of macromolecular waste that is unable to be degraded, leading to host cell death. Vorin-cat may also promote secondary infections by suppressing autophagy, since this process is also involved in killing intracellular pathogens [95]. Vorin-cat shares a 29% sequence identity with the mART domain of the CARDS toxin from *Mycoplasma pneumoniae*, which, in addition to activating the NLRP3 inflammasome, has been shown to induce formation of late endosome-derived vacuoles [96,97]. The vacuolating activity of this mART homolog helps set an interesting precedent for the possibility that Vorin-cat modulates endosomal or autophagy-related pathways. Whether the reduction of VPS15 protein occurs indirectly due to another effect of Vorin-cat, or directly via inhibition of VPS15 translation, would still need to be determined. Furthermore, the role of Vorin-cat in modulating autophagy is only worthy of consideration if it is secreted as an anti-plant cell effector and not a bactericidal toxin, as autophagy is a conserved process in plants, but not in prokaryotes [98,99].

Protein TIM11, also known as ATP synthase subunit e, exhibited a 2.3-fold negative enrichment. ATP synthase subunit e promotes ATP synthase oligomerization in the inner mitochondrial membrane, where dimerization enables local membrane curvature and the formation of cristae, which increase the mitochondrial membrane surface area to maximize ATP production [100,101]. A reduction in the amount of the TIM11/subunit e caused by Vorin-cat would presumably hinder mitochondrial membrane curvature, thus placing an energetic burden on the host cell by limiting ATP production.

Protein FMP42 was 1.9-fold negatively enriched following expression of Vorin-cat. This protein does not have an experimentally confirmed function. However, Protein FMP42 was found to be highly purified in high-throughput yeast proteomic studies and was shown to interact with the yeast protein Atg27p [102,103]. During autophagy, Atg27p localizes to the vacuolar membrane from the trans-Golgi network via the ALP pathway, a transport pathway independent of the VPS15-regulated CPY pathway [91,104]. Atg27p was shown to be important for the correct sorting of Atg9, a functionally uncharacterized protein that is essential for autophagy [104,105]. In addition to significantly reducing cellular levels of VPS15, expression of Vorin-cat in yeast appears to further impair autophagic processes by preventing synthesis of Atg27p.

The ALP pathway involves the adaptor protein AP-3, which, unlike the CPY pathway, transports cargo from the trans-Golgi network to the vacuole in a manner that circumvents the late endosome [106]. Interestingly, AP-3 subunit sigma displayed a 1.9-fold positive enrichment following induction of Vorin-cat expression, which was an enrichment opposite from that observed with FMP42, but equal in magnitude. It is possible that the observed increase in this AP-3 subunit is a secondary effect of Vorin-cat activity, as this increase may be a compensatory mechanism used by the cell to maintain normal autophagic vacuolar cargo trafficking in response to decreased levels of VPS15 and FMP42.

The protein that showed the greatest increase in abundance following induction of Vorin-cat expression was the sphingosine N-acyltransferase, LAC1, which exhibited a 2.7-fold positive enrichment. LAC1 is a crucial enzyme in sphingolipid metabolism, as it is a ceramide synthase [107]. Ceramide is an inducer of autophagy; it is an activator of JNK1, which phosphorylates Bcl-2, causing it to dissociate from BECN1, which in turn attenuates repression of autophagy by freeing up BECN1 to interact with VPS34 [90,108,109]. If Vorin-cat does, in fact, act to suppress autophagy by preventing PI3P production, an increase in ceramide biosynthesis could justifiably compensate for a decrease in autophagy initiation signals. Interestingly, the principal virulence factor of *E. amylovora*, DspA/E, arrests yeast cell growth by depleting long chain bases (LCBs), which are precursors of ceramides [110]. If DspA/E and Vorin-cat were to be secreted in tandem, they could conceivably exert a synergistic effect whereby both virulence factors act to suppress autophagy by eliminating initiation signals: Vorin-cat depletes VPS15 to impede synthesis of PI3P by VPS34, and DspA/E depletes LCBs to inhibit ceramide synthesis.

The last three proteins all increased in abundance upon Vorin-cat expression, but their enrichment did not provide any clear insight into cellular pathways that may be affected during pathogenesis. Proteins BOI1/BOB1, DEG1, and DSE4 showed 2.4-fold, 1.7-fold, and 1.2-fold increases in abundance compared to the control sample set, respectively. BOB1 is essential for yeast budding and for vesicle exocytosis during polarized cell growth [111]. DEG1 is a tRNA pseudouridine (38/39) synthase that forms pseudouridines in tRNA anticodon loops [112]. DSE4 is an endo-1,3(4)-beta-glucanase involved in cell separation after cytokinesis by dissolving the cell septum [113].

Half (4/8) of the significantly, differentially enriched proteins suggested that Vorin-cat targets a substrate with a crucial role in the regulation of autophagy. However, besides transcriptional repression caused by ADP-ribosylation of DNA sequences encoding the differentially enriched yeast proteins presented above, it is unclear what singular substrate may be targeted by Vorin-cat to cause all the observed effects based on these results alone. As the DNA-targeting mARTs, Scabin and Pierisin-1, are among the closest homologs of Vorin, the target substrate of Vorin-cat may, in fact, be a nucleotide base, or a specific nucleotide motif that affects transcription of genes encoding the proteins with significantly altered enrichments. Identification of ADP-ribosylated targets via mass spectrometry analysis may be useful in identifying proteins that show a mass increase of 542 Da, which is the mass of ADP-ribose after the loss of a hydroxide ion, if the macromolecular target is, in fact, proteinaceous.

### 2.11. Expression of Vorin-Cat Requires Co-Expression with Its Cognate Immunity Protein VorinI

**Figure 7:**
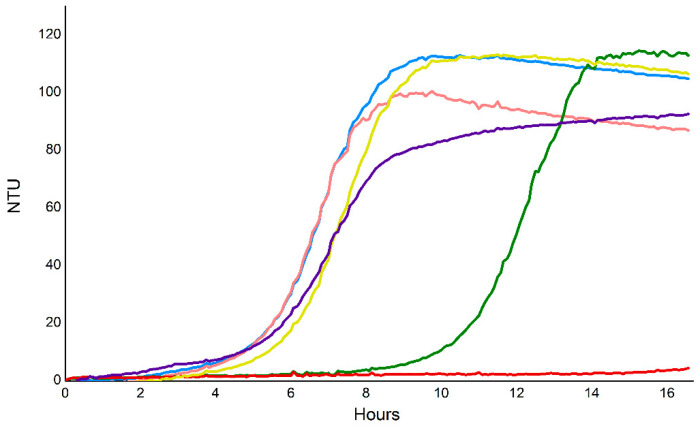
Growth curves of *E. coli* BL21(DE3) cultures expressing Vorin-cat and/or VorinI. NTU = nephelometric turbidity units. Values for wells containing sterile media blanks were subtracted from each NTU value. Empty vector (blue), empty vector with isopropyl β-d-1-thiogalactoside (IPTG) (pink), Vorin-cat (green), Vorin-cat with IPTG (red), Vorin-cat and VorinI (yellow), Vorin-cat and VorinI with IPTG (purple).

Multiple over-expression and immobilized-metal-affinity chromatography (IMAC) purification attempts of Vorin-cat WT were made using various cell lines, buffers, varying induction times and varying temperatures without success, while catalytic variants produced modest over-expression, but could only be recovered from insoluble fractions (data not shown). The fact that only catalytic variants of Vorin-cat could be over-expressed suggested that a significant amount of cell death occurred upon expression of wild-type toxin. An *E. coli* growth curve experiment was performed to investigate whether IMAC-purification of Vorin-cat WT from *E. coli* was not possible due to its potent toxic effect (Figure 7). *E. coli* cells were transformed with empty vector, transformed with a Vorin-cat pET expression vector, or co-transformed so that both Vorin-cat and VorinI would be expressed.

*E. coli* cultures expressing Vorin-cat exhibited a significant growth defect as no cell growth was detectable for the entire course of the assay. Cells that were transformed with the Vorin-cat plasmid, but not induced, displayed an appreciable growth lag as they reached stationary phase six hours later than the uninduced empty vector control culture. Induced empty vector control cultures showed slightly impaired growth compared to the uninduced control cultures. Intriguingly, co-expression of VorinI with Vorin-cat alleviated the bacteriostatic effect observed for cultures expressing only Vorin-cat. Likewise, *E. coli* cells that were co-transformed with both expression vectors encoding Vorin-cat and VorinI, but where expression was not induced, exhibited viable growth that was comparable to the empty vector, uninduced control cultures. In fact, uninduced Vorin-cat/VorinI cultures reached a higher cell density than induced, empty vector control cultures. The difference in maximum cell density/turbidity achieved by induced and uninduced empty vector control cultures was not totally unexpected; this effect could be attributed to the bioenergetics commitment required for the over-expression of the exogenous protein species encoded by the unaltered vector MCS. Growth curves of *E. coli* cultures expressing Vorin-cat WT confirmed that Vorin-cat elicits a growth defect when expressed in not only a eukaryotic model, but also in a bacterial model. This finding corroborated the challenge associated with over-expression of the toxin. A live/dead cell viability assay determined that the observed growth deficient phenotype was due to the bacteriostatic activity of Vorin-cat. Given the complete set of T6SS homologs encoded upstream of Vorin, and the involvement of T6SS effectors in a prokaryotic antagonism, Vorin may represent a T6SS-secreted effector that modulates intraspecies competition to confer a selective advantage rather than by directly infecting a *Rubus* genus plant host.

**Figure 8:**
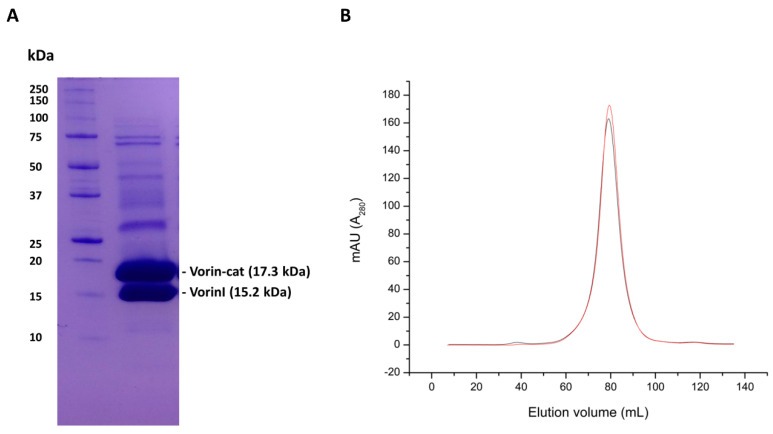
Recombinant wild-type Vorin-cat and VorinI complex. (**A**) Vorin-cat and VorinI separated by sodium dodecyl sulfate polyacrylamide gel electrophoresis (SDS-PAGE) and stained with Coomassie Brilliant Blue after gel filtration purification. (B) Gel-filtration chromatogram of co-purified Vorin-cat and VorinI. Overlapping black and red peaks correspond to two identical gel filtration runs loaded with 5 mg of IMAC-purified Vorin-cat and VorinI.

Following confirmation that Vorin-cat was too toxic to *E. coli* BL21(DE3) cells for heterologous expression, and that VorinI was able to restore growth of cells expressing Vorin-cat, Vorin-cat was co-expressed with VorinI for nickel immobilized-metal-affinity chromatography purification, followed by gel filtration purification. Unlike with Vorin-cat catalytic variants, co-expression with VorinI resulted in soluble expression of Vorin-cat and allowed for the purification of wild-type Vorin-cat protein, which was not possible without VorinI. Bands were present at 17.3 kDa and 15.2 kDa, indicating that both Vorin-cat and VorinI, respectively, were successfully purified (Figure 8A). Even though only Vorin-cat possessed a hexahistidine tag, both bands were detectable at approximately equivalent intensities. Homogeneity was not achieved as several other high molecular weight bands were detectable. During gel-filtration chromatography purification, Vorin-cat and VorinI eluted as a single, major peak, proving that they bind to form a complex (Figure 8B). Interestingly, all other protein species present in the purified sample would also have eluted as part of this peak, since no minor peaks were present. These other proteins, corresponding to lower intensity bands in Figure 8A, could potentially represent different oligomeric species of Vorin-cat and/or VorinI. If this is the case, the binding interactions would have high affinity as they did not appear to be disrupted on a denaturing sodium dodecyl sulfate polyacrylamide gel electrophoresis (SDS-PAGE) gel. Conversely, the bands could also be contaminants that were tightly bound to either Vorin-cat or VorinI during purification.

These results suggest that the two proteins interact in solution and form a complex in a manner that blocks the enzymatic activity of Vorin-cat. Given that the band intensities for both proteins were very similar, it is likely that Vorin-cat and VorinI interact in a 1:1 ratio; this ratio is further supported by the identical residue length of each protein (133 residues). As hypothesized based on the genomic organization of the Rhs1-RhsI1 TA module from *S. marcescens* Db10, and as evidenced by its restorative effect on the viability of the tested *E. coli* cultures, VorinI is indeed the cognate immunity protein of Vorin. The possibility that Vorin is a bactericidal toxin is further supported by the presence of a requisite immunity protein, since *E. amylovora* ATCC BAA-2158 would only require a protective mechanism against autointoxication if the molecular target of Vorin was conserved, which is more likely the case for prokaryotic antagonism than if Vorin targeted a plant cell macromolecule for ADP-ribosylation.

## 3. Conclusions

The discovery of secreted virulence factors advances our understanding of how bacterial pathogens cause disease in their hosts and, consequently, allows us to exploit the arsenal of toxins they produce to disarm deadly pathogens. More specifically, the discovery of each new mART toxin strengthens our understanding of the mechanisms by which these toxins enter host cells, and potentially expands the list of target substrate macromolecules that can be ADP-ribosylated. The continued study of new mART toxins also unveils new targets for the development of inhibitory anti-virulence compounds as an alternative to antibiotics in the face of declining antimicrobial efficacy [5,41,114,115].

In this work, five new putative mART toxins have been introduced, and the preliminary characterization of one bona fide mART toxin, Vorin, was described. The bioinformatics techniques used to discover them was briefly described, and the ability of Vorin and Garvin to elicit a cytotoxic effect in a eukaryotic host was confirmed. Notably, their toxic effect was shown to be due to their enzymatic activity as catalytic variants failed to cause a growth-deficient phenotype in the yeast cell host tested. Four putative toxins that were discovered using the same bioinformatics strategy were also tested in the yeast growth deficiency assay but failed to elicit any observable toxic effect. Before being ruled out as new mART toxins, these putative toxins should be assayed for cytotoxicity potential in the natural host of the bacteria that encode them, or in model cell lines.

The protein sequence of Vorin showed characteristics typical of the Rhs family of proteins. Also consistent with several Rhs proteins, Vorin was proposed to be secreted via a T6SS upon inspection of the surrounding genomic neighbourhood as encoded by *E. amylovora* ATCC BAA-2158. Vorin was also proposed to be a new member of the CT-like grouping of mART toxins based on shared sequence identity and phylogenetic relationship with characterized CT-like mART toxins, and with members of the DNA-targeting group of mART toxins, which are also CT-like group homologs. Quantitative mass spectrometry experiments revealed that Vorin-cat may play a role in suppressing host cell autophagy, which would make it the first mART toxin to inflict damage on host cells in this manner.

Growth-curve experiments were performed in *E. coli* where Vorin-cat was expressed with and without a putative immunity protein. Expression of Vorin-cat WT resulted in significant culture growth defects, while co-expression with the immunity protein, VorinI, granted a restorative effect. Recombinant Vorin-cat was successfully co-purified with its cognate immunity protein VorinI. The two proteins were shown to interact in solution, as both proteins were purified by IMAC when only Vorin-cat contained a hexahistidine tag. Purification of Vorin-cat and VorinI by gel filtration chromatography demonstrated that both proteins eluted as a single, distinct peak. These findings confirmed that VorinI is the antitoxin that confers immunity to Vorin-cat.

The principal focus of immediate future work should be to solve the three-dimensional structure of the Vorin-cat/VorinI complex by X-ray crystallography as no toxin-antitoxin pair has ever been reported for secreted mART toxins. Structural data would uncover the molecular basis for the inhibition of Vorin-cat, and by extension, may provide invaluable structural information on how to synthetically occlude the conserved mART active site in order to inhibit other potent CT-like mART toxins such as cholera toxin or pertussis toxin. The natural substrate of Vorin-cat should also be determined to elucidate the molecular basis for the observed growth defects. Quantitative mass spectrometry suggested that Vorin-cat may play a role in suppressing autophagy in host cells, possibly leading to cell death via an accumulation of autophagic substrates. Despite this finding, the mechanistic basis for the considerable toxicity observed in *E. coli* is still unclear since autophagy is not a conserved process in bacteria. The toxic mechanism of Vorin-cat upon expression in bacteria should thus be examined by repeating the experiment using *E. coli* as the expression host.

The research presented in this study revealed that Vorin possesses features uncharacteristic of previously discovered mARTs. Most importantly, Vorin was revealed to be, to our knowledge, the first mART toxin with an immunity binding partner while also likely being deployed by a T6SS. Two other mART toxins with cognate antitoxins, DarT and ParT, have recently been described, but these mARTs are intracellular mediators of bacteriostasis, and not secreted virulence factors [116,117]. While Vorin-cat was also shown to exhibit bacteriostatic activity, the proximity to a T6SS gene cluster suggests that Vorin may have initially been a similar intra-cellular effector to DarT and ParT before being repurposed as a secreted virulence factor. The implications of this bacteriostatic activity remain to be elucidated, however, since it is unclear whether Vorin-cat is deployed as an effector that mediates intra- or inter-species competition, or if the natural substrate is simply conserved between bacteria and eukaryotes. Nonetheless, Vorin is the newest member of the ADP-ribosyltransferase family of toxins. The continued characterization of Vorin could yield treatment options against fire blight by further uncovering an exploitable toxin mechanism responsible for its progression.

## 4. Materials and Methods

### 4.1. Database Search for Candidate mART Toxin Sequences and Sequence Processing

Retrieval of all new mART toxin candidate sequences was performed using the remote homology search tools HHsenser and HHblits (MPI Bioinformatics Toolkit, Max Planck Institute for Developmental Biology, Tübingen, Germany) [35,36]. A minimum E-value threshold of 1 × 10^−3^ was set with a maximum of 3 iterations, and the number of target sequences was set to 1000. The catalytic domains of every mART toxin belonging to the C2-like and C3-like subgroups as defined in Simon et al. (2014) and, additionally, the catalytic domains of VahC, C3larvin and Plx2A toxins, were used as input for HHsenser and HHblits searches [3]. Remote homolog sequences that were longer than 1000 amino acid residues that exhibited a sequence identity greater than 50% to any sequence from a custom database of all known mART toxins, and duplicate sequences, were discarded. The ScanProsite tool (SIB ExPASy Resource Portal) was used to filter remote homologs for those containing the characteristic mART toxin regular expression [YFL]-R-x(27,60)-[YF]-x-S-T-[SQT]-x(32,78)-[QE]-x-E [30,118]. The output data were sorted in Excel to isolate UniProt identifiers for sequences that matched the regular expression. The webservers SignalP 4.1 and SecretomeP 2.0 were used to conduct protein secretion predictions, either via signal peptide or non-classical secretion [119,120]. Sequences scoring higher than the recommended thresholds of 0.45 for SignalP and 0.5 for SecretomeP were retained for further analysis. By default, sequences that scored higher than the 0.45 threshold were also predicted to contain no transmembrane domains. Phobius (Stockholm Bioinformatics Centre) was used as an additional predictor of protein secretion for sequences originating from “Gram-variable” bacteria; default settings were used, and sequences predicted to be extracellular and lacking transmembrane domains were retained [121]. Sequencing data submitted to GenBank/NCBI was manually inspected to examine the genomic context of each candidate coding sequence. UniProt BLAST searches were used to identify gene products indicative of pathogenesis such as secretion systems, transposons, or accessory toxins. Candidates with limited genomic context information were discarded due to uncertainty. The bacterial species encoding all retained sequences were classified as relevant pathogens by performing manual literature searches. Non-pathogen sequences were discarded. Multiple sequence alignments were performed with T-COFFEE using the “Expresso” structural alignment option. Phylogenetic trees were created using Phylogeny.fr by Bayesian inference [122]. Default settings were otherwise used, except a T-COFFEE alignment was selected with no alignment curation.

Protein structural models were created using RaptorX [123]. Models with unnormalized and normalized global distance test scores (uGDT and GDT, respectively) greater than 50, and with *p*-value scores less than 1 × 10^−4^, were deemed accurate. The uGDT and GDT scores indicate the global structural quality of a predicted model, while the *P*-value is the probability that a predicted model is less accurate than a set of randomly generated models [123]. Consensus-based structure predictions were performed using LOMETS [124]. Candidates that resulted in at least 7 out of 10 server predictions with a “HIGH” model score, with template coverage of at least 70% for the predicted catalytic domain as determined by RaptorX, were kept. Models were refined using FG-MD to simulate native structures [125]. Local model geometry quality was assessed using MolProbity, and global model quality was assessed using ProQ2 and ModFOLD6 [126,127,128]. Global model quality was considered acceptable with combined ProQ2 and ModFOLD6 scores of greater than 0.4, and a ModFOLD6 *p*-value of less than 1 × 10^−3^. Model images were created using the PyMOL Molecular Graphics System v1.8.2.0 (Schrödinger, LLC, New York, NY, USA).

### 4.2. Bacterial Genomic DNA

Genomic DNA (gDNA) encoding the putative mART toxins described in this study was generously donated by various researchers from around the world. *Erwinia amylovora* strain ATCC BAA-2158 (16-5) heat-treated cells were provided by Dr. Fabio Rezzonico from the Zurich University of Applied Sciences, and gDNA was extracted from the cells with the Purelink Microbiome DNA Purification Kit from ThermoFisher according to the manufacturer’s “Microbial Culture” protocol. *Bartonella bovis* strain 91-4 gDNA was provided by Kristina Naslund and Dr. Siv Andersson from Uppsala University. *Spiroplasma melliferum* strain KC3 gDNA was provided by Dr. Vladimir Babenko from the Institute of Cytology and Genetics in Novosibirsk, Russia. The *garvin* gene of interest from *Lactococcus garvieae* strain 21881 containing flanking ends homologous to the yeast expression vector pRS415-*CUP1* was synthesized by polymerase chain reaction (PCR) and sent by Dra. Alicia Gibello from The Complutense University of Madrid.

### 4.3. Transformation of Saccharomyces Cerevisiae and Yeast Growth Deficiency Assay

Putative toxin genes were amplified by PCR using primers complementary to the 5′ and 3′ ends of the gene of interest, and which contained the following sequences homologous to the pRS415-*CUP1* plasmid: CGAATTCCTGCAGCCCGGGGGATCCACTAGTTCTAGA (5′-end of forward primer) and CAGTTAGCTAGCTGAGCTCGAGACGGCCGCTCTAGA (3′-end of reverse primer). Reaction mixtures contained 5 ng purified gDNA, 5 pmol of forward and reverse primer, 10 nmol of dNTPs and 1-unit Phusion Hot Start II DNA Polymerase (Thermo Scientific) in 50 µL 1X Phusion HF Buffer with MgCl_2_ added to a final concentration of 2.5 mM. Cycling conditions consisted of an initial denaturation at 98 °C for 3 min, 30 cycles of 98 °C for 30 s, annealing at the appropriate temperature for 30 s, and extension at 72 °C for 30 s, followed by a final extension at 72 °C for 5 min. Successful amplification was confirmed by gel electrophoresis. Amplicons were purified using the GeneJET PCR Purification Kit (Thermo Scientific) according to manufacturer’s instructions. The centromeric plasmid pRS415 with a Cu^2+^-inducible *CUP1* promoter, and the principle of in vivo recombination with a PCR-amplified gene insert, were used for all yeast growth deficiency assays transformations as previously described [37]. Electrocompetent *Saccharomyces cerevisiae* W303 (*MAT*a*/MATα, leu2-3112 trp1-1 can1-100 ura3-1 ade2-1 his3-11,15*) cells were prepared based on a modified protocol [129]. Ten microlitres of electrocompetent cells were combined with 40 µL ice-cold 1 M D-sorbitol, 180 ng digested pRS415-*CUP1* and 60 ng of the desired PCR-amplified gene insert or only undigested pRS415-*CUP1* and placed on ice for 5 min. The cell-DNA mixture was pipetted into a 1 mm Fisherbrand™ Electroporation Cuvette Plus™ and electroporated at 2.5 kV using a Bio-Rad MicroPulser™ electroporator. Cells were recovered in 8 mL yeast peptone dextrose (YPD) containing 0.15 mg/mL adenine and 0.5 M D-sorbitol at 30 °C for 1 h with shaking. Cells were harvested by centrifugation at 1500× *g*, resuspended in approximately 400 µL of the supernatant, and plated on synthetic-defined agar medium lacking leucine (SD-LEU) containing 1M D-sorbitol, 0.15 mg/mL adenine, and 100 µg/mL ampicillin. Plates were incubated at 30°C for 72 h. Tubes of 5 mL SD-LEU containing 0.15 mg/mL adenine and 100 µg/mL ampicillin were inoculated with transformant yeast colonies from either control or experimental toxin gene transformations and incubated at 30 °C for 18 h with shaking. Culture OD (595 nm) readings were taken after 18 h, and the values were used to dilute each culture to OD_595_ = 2 × 10^−4^ in sterile 1.5 mL tubes with fresh SD-LEU containing the same concentrations of adenine and ampicillin. Expression of control and putative mART toxins was induced by addition of CuSO_4_ to a final concentration of 0 mM, 0.25 mM, 0.5 mM or 0.75 mM. Each control or experimental culture was dispensed into a 96-well plate in 100 µL aliquots such that each concentration of CuSO_4_ was repeated in 6 biological replicates, each consisting of 4 technical replicates. The 96-well plates were sealed and placed at 30 °C for 48 h. Plates were shaken vigorously for one minute, and absorbance measurements at 595 nm were taken of each well using a FLUOstar^®^ Omega microplate reader (BMG LABTECH).

### 4.4. Site-Directed Mutants of Vorin-Cat and Garvin

All mutations resulting in catalytic residue substitutions were performed using the QuikChange^®^ Mutagenesis method (Stratagene, San Diego, CA, USA) according to the manufacturer’s protocol. The Glu residues of the E-x-E motifs of Vorin-cat and Garvin were substituted to Ala residue (A-x-A) double variants in a single reaction. Successfully mutated plasmid DNA was confirmed by next-generation sequencing using an Illumina MiSeq^®^ system at the Advanced Analysis Centre at the University of Guelph.

### 4.5. Preparation of Yeast Cell Protein for LC-MS/MS

*S. cerevisiae* BY4741 (*MATa, his3Δ1 leu2Δ0 met15Δ0 ura3Δ0*) was transformed with *Xba*I-digested pRS415-*CUP1* and PCR-amplified in vivo recombination (IVR) vorin-cat by electroporation and plated on YPD agar containing 0.15 mg/mL adenine and 100 µg/mL ampicillin. Plates were incubated at 30 °C for 3 days. Single colonies were used to inoculate 6 tubes of 5 mL SD-LEU cultures containing 0.15 mg/mL adenine and 100 µg/mL ampicillin. Cultures were incubated at 30 °C with shaking until OD_595_ = 0.4 (~10^7^ cells). In three of the tubes, expression of Vorin-cat was induced by adding 0.1 mM CuSO_4_ at the time of colony inoculation, while the other three cultures were not induced. Cell pellets were harvested by centrifugation at 2040 x *g* for 5 min. Cells were washed twice with phosphate-buffered saline (PBS), and centrifuged at 300× *g* for 5 min at 0 °C. Cells were resuspended in 100 mM Tris pH 7.6, 10 mM dithiothreitol 4% SDS, and sonicated in a 4 °C water bath for 15 min (30 s on, 30 s off). Cell lysates were incubated at 95 °C for 10 min with agitation, cooled to room temperature, combined with 55 mM iodoacetamide, and incubated in the dark for 20 min. Acetone was added to a final concentration of 80%, and lysates were incubated overnight at −20 °C. Samples were centrifuged at 18,000× *g* for 20 min at 0 °C and washed twice with 80% acetone. Pellets were dried at 40 °C for 15 min, and resuspended in 8 M urea, 10 mM HEPES. Samples were digested with LysC and trypsin proteases at a protease-to-sample protein ratio of 1:100 at room temperature overnight. Total sample was loaded onto C_18_ STAGE-tips (EmporeTM, IVA-Analysentechnik, Meerbusch, Germany). Liquid chromatography was performed using an EASY-nLC system (Thermo Fisher Scientific, Bremen, Germany). Mass spectrometry analysis was accomplished using a Q Exactive HF quadrupole orbitrap mass spectrometer (Thermo Fisher Scientific, Mississauga, ON, USA). Peptides were separated over a 60-min linear gradient on a reverse-phase 50 cm C_18_ column packed with 3 µm silica beads (ReproSil-Pur C_18_-AQ, Dr. Maisch GmbH, Ammerbuch-Entringen, Germany).

*S. cerevisiae* BY4741 (*MATa, his3Δ1 leu2Δ0 met15Δ0 ura3Δ0*) was transformed with *Xba*I-digested pRS415-*CUP1* and PCR-amplified in vivo recombination (IVR) vorin-cat by electroporation and plated on YPD agar containing 0.15 mg/mL adenine and 100 µg/mL ampicillin. Plates were incubated at 30 °C for 3 days. Single colonies were used to inoculate 6 tubes of 5 mL SD-LEU cultures containing 0.15 mg/mL adenine and 100 µg/mL ampicillin. Cultures were incubated at 30 °C with shaking until OD_595_ = 0.4 (~10^7^ cells). In three of the tubes, expression of Vorin-cat was induced by adding 0.1 mM CuSO_4_ at the time of colony inoculation, while the other three cultures were not induced. Cell pellets were harvested by centrifugation at 2040× *g* for 5 min. Cells were washed twice with phosphate-buffered saline (PBS), and centrifuged at 300× *g* for 5 min at 0°C. Cells were resuspended in 100 mM Tris pH 7.6, 10 mM dithiothreitol 4% SDS, and sonicated in a 4°C water bath for 15 min (30 s on, 30 s off). Cell lysates were incubated at 95 °C for 10 min with agitation, cooled to room temperature, combined with 55 mM iodoacetamide, and incubated in the dark for 20 min. Acetone was added to a final concentration of 80%, and lysates were incubated overnight at −20 °C. Samples were centrifuged at 18,000× *g* for 20 min at 0 °C and washed twice with 80% acetone. Pellets were dried at 40 °C for 15 min, and resuspended in 8 M urea, 10 mM HEPES. Samples were digested with LysC and trypsin proteases at a protease-to-sample protein ratio of 1:100 at room temperature overnight. Total sample was loaded onto C_18_ STAGE-tips (EmporeTM, IVA-Analysentechnik, Meerbusch, Germany). Liquid chromatography was performed using an EASY-nLC system (Thermo Fisher Scientific, Bremen, Germany). Mass spectrometry analysis was accomplished using a Q Exactive HF quadrupole orbitrap mass spectrometer (Thermo Fisher Scientific, Mississauga, ON, USA). Peptides were separated over a 60-min linear gradient on a reverse-phase 50 cm C_18_ column packed with 3 µm silica beads (ReproSil-Pur C_18_-AQ, Dr. Maisch GmbH, Ammerbuch-Entringen, Germany).

### 4.6. LC-MS/MS Data Analysis

Liquid chromatography–tandem mass spectrometry (LC-MS/MS) spectra were processed using the MaxQuant v1.6.2.10 software package [130]. Quantification was performed with the built-in label-free quantification (LFQ) algorithm, with “match between runs” selected. The Andromeda search engine was used to search the spectra against the UniProt *S. cerevisiae* s288c proteome (taxonomy ID 559292, 9 October 2018). Statistical analyses of LC-MS/MS data were performed with the software platform Perseus v1.6.2.2 [131]. Data were filtered for protein contaminants and for artifactual reverse peptides. Data were log_2_-transformed, and missing values were replaced by values estimated from a normal distribution. Only proteins present in duplicate (n = 2) in both uninduced and induced sample sets were used for further analysis. A two-sample Student’s *t*-test (*p* value < 0.05) was performed to identify proteins that significantly differed in abundance between both sample sets using a 5% false discovery rate (FDR) control filter.

### 4.7. Transformation, Expression and Purification of Vorin-Cat and VorinI

The *vorin-cat* gene was cloned into a pET-TEV vector with an N-terminal hexahistidine tag. The *vorinI* gene was cloned into a pCDFDuet-1 vector with no expression tag. Chemo-competent *Escherichia coli* BL21(DE3) was co-transformed with both plasmids by heat shock method. Cells were grown in 4L 2xYT media containing 30 µg/mL kanamycin and 100 µg/mL streptomycin at 37 °C with shaking until OD_600_ = 0.6, and protein expression was induced with 0.1 mM IPTG for 18 h at 16 °C with shaking. Cells were harvested by centrifugation at 3750× *g* for 15 min and resuspended in lysis buffer (50 mM Tris pH 7.5, 250 mM NaCl, 10% glycerol). Cells were lysed using an Emulsiflex C3 microfluidizer (Avestin, Ottawa, ON, Canada) in the presence of 20 µg/mL CHAPS, 2 mM EDTA, 100 µg/mL DNase and 0.8 mM PMSF. Cell lysate was centrifuged at 23,600× *g* for 55 min at 4 °C. The supernatant was mixed with 20 mM MgCl_2_ for 30 min at 4°C. One millilitre of Chelating Sepharose^®^ Fast Flow resin (GE Healthcare, Mississauga, ON, Canada) charged with Ni^2+^ ions was then added, and mixed for another 30 min at 4 °C. The entire mixture was loaded into an Econo-Pac^®^ chromatography column (Bio-Rad, Mississauga, ON, Canada) and the lysate containing unbound protein was eluted. The resin was then washed with one column volume (~25 mL) lysis buffer (50 mM Tris pH 7.5, 250 mM NaCl, 10% glycerol), one column volume lysis buffer containing 10 mM imidazole and one column volume lysis buffer containing 25 mM imidazole. His_6_-tagged Vorin-cat/VorinI was eluted with one column volume of lysis buffer containing 250 mM imidazole. The purity of fractions containing eluted His_6_-tagged Vorin-cat/VorinI was assessed by SDS-PAGE, and fractions containing Vorin-cat/VorinI were pooled and dialyzed overnight against 50 mM Tris pH 7.5, 250 mM NaCl, 10% glycerol. Vorin-cat/Vorin was further purified by gel filtration chromatography using a HiLoad 16/60 Superdex-200 column (GE Healthcare), and fractions showing the presence of Vorin-cat and VorinI after separation by SDS-PAGE were pooled and concentrated.

### 4.8. E. coli Cell Viability Assays

*E. coli* BL21(DE3) cells were transformed with 100 ng of the appropriate expression vector or combination of vectors by heat shock method, and a single colony was used to inoculate a 5 mL LB culture containing either 30 µg/mL kanamycin or 30 µg/mL kanamycin and 100 µg/mL streptomycin. The 5 mL cultures were incubated at 37 °C for 6 h with shaking. Culture OD_600_ readings were taken after 18 h, and the values were used to dilute each culture to OD_600_ = 2 × 10^−4^ in sterile 1.5 mL tubes with fresh LB containing the appropriate antibiotic. Expression of Vorin-cat and VorinI was induced by addition of IPTG to a final concentration of 0.1 mM. Each control or experimental culture was dispensed into a 96-well plate in 200 µL aliquots. Plates were incubated at 35°C with shaking using a NEPHELOstar nephelometer (BMG LABTECH) and culture turbidity was measured in nephelometric turbidity units (NTU) every 5 min for 16.5 h. The BacLight Live/Dead bacterial viability kit (L-7012, Molecular Probes) was used to assess cell viability after a three-hour induction of *vorin-cat* in *E. coli* Lemo21 cells.

## Figures and Tables

**Table 1:** LOMETS consensus prediction scores.

Organism ^1^	UniProt Sequence Identifier	Average Template Coverage	Server Predictions with HIGH Confidence (Out of 10)	Overall Confidence
*Spiroplasma* *melliferum*	A0A037UQ33	0.857	10	High
*Weissella* *cibaria*	A0A139R9U4	0.955	10	High
*Parachlamydia acanthamoebae*	A0A0C1E5A0	0.901	10	High
*Lactobacillus* *kunkeei*	A0A0C3AG37	0.854	10	High
*Escherichia* *coli*	T9AJP6	0.914	10	High
*Fusobacterium necrophorum*	A0A064A9T9	0.948	10	High
*Pectobacterium carotovorum*	A0A093SYU3	0.701	6	Medium
*Legionella* *quateirensis*	A0A0W0XJR5	0.708	10	Medium
*Enterobacter* *bugandensis*	A0A167JVD8	0.857	7	High
*Photobacterium damselae*	D0Z4Y1	0.660	7	Medium
*Providencia* *alcalifaciens*	W3YC38	0.864	10	High
*Erwinia* *amylovora*	E5B8T9	0.510	10	Medium
*Paenibacillus* *popilliae*	M9M5D5	0.839	10	High
*Bartonella* *bovis*	N6VGJ3	0.831	10	High
*Pantoea* *stuartii*	E0LU50	0.671	3	Medium
*Lactococcus* *garvieae*	H2B2R7	0.812	10	High

^1^ The organism is the bacterial species that produces each protein, while the UniProt identifier is the accession ID for each specific protein sequence. Template coverage is the average fraction of residues considered for each server’s prediction out of 1.0. The overall confidence is a final consensus score given by the LOMETS server which consolidates average template coverage with the number of high confidence server predictions.

**Table 2:** RaptorX threading prediction scores.

Organism	UniProt Sequence Identifier	Residues ^1^ Modelled (mART Domain)	*p*-Value	uGDT (GDT)	uSeqID (SeqID)	Templates
*Spiroplasma melliferum*	A0A037UQ33	1–261	4.83 × 10^−9^	169(65)	97(37)	4fxqA 4fk7A
*Weissella* *cibaria*	A0A139R9U4	1–188	6.44 × 10^−7^	131(70)	38(20)	1qs2A 1qs1A 5gttA 2j3zA 4fxqA
*Parachlamydia acanthamoebae*	A0A0C1E5A0	42–250	7.73 × 10^−6^	126(51)	53(21)	4xzjA 1qs2A 1qs1A
*Lactobacillus kunkeei*	A0A0C3AG37	32–237	2.29 × 10^−7^	121(51)	49(21)	1qs1A 1qs2A 5gttA 4fxqA 4fk7A
*Escherichia* *coli*	T9AJP6	1–220	3.57 × 10^−7^	119(54)	32(15)	4xzjA 1qs1A 1qs2A 4elnA 4fxqA
*Fusobacterium necrophorum*	A0A064A9T9	306–475	1.58 × 10^−6^	119(70)	36(21)	4xzjA
*Legionella quateirensis*	A0A0W0XJR5	1–324	7.80 × 10^−7^	115(36)	36(11)	4xzjA 4fxqA 1qs2A 1qs1A 3bw8A
*Enterobacter bugandensis*	A0A167JVD8	735–951	1.31 × 10^−8^	105(48)	36(17)	4xzjA
*Providencia alcalifaciens*	W3YC38	102–408	1.00 × 10^−4^	102(33)	41(13)	4xzjA 1qs1A 1qs2A 5gttA 2wn4A
*Erwinia amylovora*	E5B8T9	144–276	3.67 × 10^−8^	93(73)	33(26)	1tiiA 1xtcA 1lt4A 4z9dA 5ewkA
*Paenibacillus popilliae*	M9M5D5	1–222	1.22 × 10^−6^	121(54)	42(19)	1qs1A 1qs2A 4fk7A 4fxqA 2bovB
*Bartonella* *bovis*	N6VGJ3	1–238	3.82 × 10^−12^	141(59)	68(29)	1lt4A 1tiiA 1ltiA
*Lactococcus garvieae*	H2B2R7	1–239	5.56 × 10^−7^	116(48)	37(15)	2a78B 5gttA 2bovB 3bw8A 4fxqA

^1^ Only residues corresponding to the predicted mART catalytic domain were submitted to RaptorX for structural predictions. The *p*-value is the likelihood of a model being less accurate than the highest-scoring model of a randomly-generated set. The uGDT (unnormalized Global Distance Test) is the global model quality, and the GDT is the uGDT normalized to the sequence length. The templates are listed as Protein Data Bank (PDB) structure IDs. According to Ma et al., a *p*-value less than 10^−4^, a SeqID >30%, and a uGDT for proteins with greater than 100 residues or a GDT >50 for proteins with less than 100 residues are all good measures of an accurate structural prediction.

**Table 3:** Highlights of the final six mART toxin candidates.

Putative mART Toxin	UniProt Accession	mART Homologs (% Sequence ID)	Genomic Neighbours Indicative of Virulence	Research Interest
**Vorin**	E5B8T9	Pierisin (33%), LT-A (30%) CARDS (29%), Cholera (29%) Scabin (27%)	T6SS subunit homologs, toxin-antitoxin modules	Fire blight in *Rubus* genus plants
**Garvin**	H2B2R7	Certhrax (24%) C3bot1 (24%)	Collagen- and mucin-binding proteins	Lactococcosis in salmonid fish; various zoonoses (e.g., UTIs and skin infections)
**Bovin**	N6VGJ3	LT-IIA/B (40%) Cholera toxin (36%) Pierisin (32%) Scabin (31%)	Possible B-domain subunit (similar to cholera toxin pentameric subunit)	Endocarditis in beef and dairy cattle
**Mellifin**	A0A037UQ33	Certhrax (42%) C3bot1, C3lim, C3stau1 (29%)	Protective antigen, integrase, PTS glucose transporter	Spiroplasmosis in honey bees?
**EcX**	A0A167JVD8	Mav (27%) Vis (22%)	Transposases, permease transporter	Antibiotic-resistant nosocomial infections
**Pax**	A0A0C1E5A0	EFV (29%) Vis (28%)	Mycobacterial two-component regulatory system, chemotaxis protein, LPS synthesis proteins	Community-acquired pneumonia in humans?
