# Erratum: Tremblay, O., et al. Several New Putative Bacterial ADP-Ribosyltransferase Toxins Are Revealed from In Silico Data Mining, Including the Novel Toxin Vorin, Encoded by the Fire Blight Pathogen *Erwinia amylovora*. *Toxins* 2020, *12*, 792

**DOI:** 10.3390/toxins13030229

**Published:** 2021-03-22

**Authors:** Olivier Tremblay, Zachary Thow, Jennifer Geddes-McAlister, A. Rod Merrill

**Affiliations:** Department of Molecular and Cellular Biology, University of Guelph, Guelph, ON N1G 2W1, Canada; otrembla@uoguelph.ca (O.T.); zthow@uoguelph.ca (Z.T.); jgeddesm@uoguelph.ca (J.G.-M.)

The authors wish to make the following corrections to this paper [1]:

## 1. Change in Authorship (Add One New Author)

In the original paper [1], Jennifer Geddes-McAlister was not included as an author in the published article. Jennifer Geddes-McAlister also contributed to investigation, formal analysis, supervision for this research. The corrected Author Contributions Statement appears here. The authors apologize for any inconvenience caused and state that the scientific conclusions are unaffected. The original article has been updated.

The authorship change from 

Olivier Tremblay, Zachary Thow and A. Rod Merrill * Department of Molecular and Cellular Biology, University of Guelph, Guelph, ON N1G 2W1, Canada; otrembla@uoguelph.ca (O.T.); zthow@uoguelph.ca (Z.T.)

to 

Olivier Tremblay, Zachary Thow, Jennifer Geddes-McAlister and A. Rod Merrill * Department of Molecular and Cellular Biology, University of Guelph, Guelph, ON N1G 2W1, Canada; otrembla@uoguelph.ca (O.T.); zthow@uoguelph.ca (Z.T.); jgeddesm@uoguelph.ca (J.G.-M.).

## 2. Change in Author Contributions

Conceptualization, O.T., Z.T. and A.R.M.; Funding acquisition, A.R.M.; Investigation, O.T., Z.T., J.G.-M. and A.R.M.; Formal analysis, J.G.-M.; Supervision, J.G.-M.; Writing—original draft, O.T.; Writing—review and editing, O.T., Z.T. and A.R.M. All authors have read and agreed to the published version of the manuscript.
